# Ultrasound Reversible Response Nanocarrier Based on Sodium Alginate Modified Mesoporous Silica Nanoparticles

**DOI:** 10.3389/fchem.2019.00059

**Published:** 2019-02-11

**Authors:** Xiaochong Li, Zhanhua Wang, Hesheng Xia

**Affiliations:** State Key Laboratory of Polymer Materials Engineering, Polymer Research Institute, Sichuan University, Chengdu, China

**Keywords:** mesoporous silica nanoparticles, sodium alginate, reversible, ultrasound response, controlled drug delivery

## Abstract

Mesoporous silica nanoparticles (MSN) covered by polymer coatings, cross-linked by weak coordination bonds were expected to present a reversible responsiveness under on-off ultrasound stimuli. Herein, we prepared a sodium alginate (SA) modified MSN with carboxyl-calcium (COO^−^-Ca^2+^) coordination bonds in the modified layer, which could block the mesopores of MSN and effectively prevent the cargo from pre-releasing before stimulation. The coordination bonds would be destroyed under the stimulation of low intensity ultrasound (20 kHz) or high intensity focused ultrasound (HIFU, 1.1 MHz), leading to a rapid and significant cargo release, and then they could be reformed when ultrasound was turned off, resulting in an instant cargo release stopping. The reversible cleavage and reformation of this coordination bonds under on-off ultrasound stimulus were confirmed by the gel-sol transition behaviors of the SA-CaCl_2_ gels. An excellent real-time control of rhodamine B (RhB) release performance was obtained under the ultrasound stimuli. Obviously, the cargo release ratio could reach to nearly 40% when HIFU (80 W) was turned on for 5 min, and remained basically constant when ultrasound was turned off, which would finally reach to nearly 100% within 30 min under this on-off pulsatile status. These hybrid MSN based nanoparticles with excellent reversible ultrasound on-off responsiveness were of great interest in on-demand drug delivery applications in the future.

## Introduction

During recent decades, with the development of nanotechnology, nanoparticles have been widely applied and well-developed in the field of biomedicine for drug delivery. The most important reason behind this revolution is that nanomaterials as drug carriers can overcome the shortcomings of the conventional small molecule drugs, such as the short half-life, the fast metabolism, and concentration reduction, which consequently decrease the therapeutic effects and increase the toxic side effects on the human body (Hubbell and Chilkoti, 2012). In fact, nanomaterials can make drugs accumulated at the tumor site and reduce the drug concentration in the normal tissue cell sites, thereby improving the anticancer effect as well as reducing the side effects (Panyam and Labhasetwar, 2003; Allen and Cullis, 2004; Farokhzad and Langer, 2009). Moreover, traditional anticancer drugs are usually very small in size and are quickly cleared in the blood so that the effective concentration at the site of the tumor will be reduced. By loading these small anticancer drugs into the appropriate nanocarriers, the circulation time of the drug molecules in the blood can be extended (Bae et al., 2011; Bogart et al., 2014).

Among various nanomaterials employed for drug delivery applications, the fast-growing attention to mesoporous silica nanoparticles (MSN) has been vastly highlighted due to their outstanding properties, such as large specific surface areas, uniform pores with adjustable structures, excellent biocompatibility, and thermal stability, as well as a facile modification on surface (Trewyn et al., 2007; Chang et al., 2013; Argyo et al., 2014; Paris et al., 2015). However, conventional bare MSN cannot effectively prevent the drugs from being pre-released because of their open mesopores, therefore they cannot achieve an accurate controlled therapeutic efficacy. One alternative that has been proposed to date is the study of the smart drug delivery system, which allows the elaborate surface modifications based on MSN and endows one or more stimuli responsiveness onto them (Chen et al., 2010; Li Z. et al., 2012; Mura et al., 2013; Argyo et al., 2014; Baeza et al., 2015; Wen et al., 2017). Smart drug delivery systems can make mesoporous entrance open or close under exposure to various single internal stimulus [i.e., pH (Li et al., 2014; Cheng et al., 2017; Zeng et al., 2017], redox substances Zhao et al., 2009; Sauer et al., 2010, and enzymes Bernardos et al., 2010; Sun et al., 2013), single external stimulus [i.e., light (Ferris et al., 2009; Martínez-Carmona et al., 2015), temperature (Chung et al., 2008; Schlossbauer et al., 2010), magnetic field (Chen et al., 2011; Xuan et al., 2018), and ultrasound (Chen Y. et al., 2014; Paris et al., 2015; Anirudhan and Nair, 2018)] or a combination of multiple stimuli (Chen X. et al., 2014; Li et al., 2015, 2018). Among all of the studied stimuli, ultrasound attracts great attention due to its robust and unique advantages, especially for the high intensity focused ultrasound (HIFU). Generally, ultrasound has two kinds of categories according to the frequency: low intensity ultrasound (20–200 kHz) and HIFU (>200 kHz). Both types of ultrasound can easily realize a spatiotemporal control of drug release at the targeted location and can easily regulate the penetrate depth by tuning the frequency, power density, and duration (Mura et al., 2013; Boissenot et al., 2016). Additionally, ultrasounds are also appealing because of their non-invasiveness, non-ionizing and cost effectiveness, as well as the enhanced cell membrane permeability. More importantly, compared with low intensity ultrasound, HIFU can focus the intense ultrasonic wave in a targeted small spot, while in other areas the intensity of the wave is relatively low and can be accepted by the human body. So HIFU is effective and safe as a promising stimulus, which has been widely used in human drug delivery applications (Schroeder et al., 2009; Xia et al., 2016).

In general, ultrasound has two main effects which are thermal effect and acoustic cavitation effect; both can play a role in triggering the release of drugs from nanocarriers (Sirsi and Borden, 2014; Xia et al., 2016). When acoustic waves are passing through a tissue the attenuation is generated. The attenuation energy will be converted into thermal effect. Acoustic cavitation is a process where plenty of microbubbles form, grow and collapse during a very short period of time when ultrasound is applied. Cavitation threshold is easily realized by low intensity ultrasound while the thermal effect is more significant when high intensity focused ultrasound (HIFU) is used. Indeed, although both thermal effect and acoustic cavitation effect can make polymers unstable or even destroyed, the latter factor is usually considered as the main reason for ultrasound-induced drug release by bond cleavage of hybrid nanocarriers (Tong et al., 2013; Chen X. et al., 2014; Paris et al., 2015; Li et al., 2016; Anirudhan and Nair, 2018). The strong physical forces associated with the collapse of cavitation bubbles can induce some cleavages of mechano-labile bonds and scission of polymer chains, which is termed as mechano-chemistry. Therefore, for those hybrid nanocarriers that are introduced with the mechano-labile bonds, they will have ultrasonic responsiveness and the modified structure will be broken up when ultrasound is on and, eventually, the inner drugs can be released (Li et al., 2017).

Previously, there have been many studies on ultrasound-induced cleavage of some chemical bonds, such as covalent bonds (ester bond, disulfide bond, Diels-Alder linkage) and supramolecular interaction (hydrogen bond, metal coordination, electrostatic interaction, and π-π interaction) in well-designed polymers, which were applied to ultrasound responsive drug delivery (Li et al., 2010, 2018; Xuan et al., 2012; Tong et al., 2013; Liang et al., 2014) and self-healing/shape memory materials (Li G. et al., 2012; Lu et al., 2014). However, most of the covalent bond cleavages in solution are irreversible, which cannot be applied to the drug delivery field in combination with nanocarriers, and cannot effectively reserve residual drugs after ultrasound stopping. On the contrary, metal coordination, one of the supramolecular interactions, can meet the above requirements well due to its reversibility (Beck and Rowan, 2003; Vermonden et al., 2003; Paulusse and Sijbesma, 2004). It has been widely demonstrated that many metal coordination complexes could achieve reversibility under certain stimulation, such as Fe(III)-polyphenols complexes (Ju et al., 2015), Cu(II)–terpyridine complexes (Liang et al., 2014), Pd(II)-phosphane complexes (Paulusse and Sijbesma, 2004), Ca(II)-carboxyl complexes (Huebsch et al., 2014), and borate-PEGylated coordination complexes (Liu et al., 2018). Reversible ultrasonic responsive hybrid nanocarriers based on metal coordination interaction and combined with MSN are expected to be achieved and applied in the field of drug delivery. To this aim, dynamic metal coordination bonds formed between carboxyl groups (COO^−^) and calcium ions (Ca^2+^) come into our view. The labile metal coordination bonds, formed by Ca^2+^ and COO^−^ of SA, may be cleavable when ultrasound is turned on and also can be reformed after ultrasound is turned off. Porous nanoparticles covered by cross-linked polymer coatings through coordination bonds formed between Ca^2+^ and COO^−^ could successfully open the pores and release the loaded cargoes by breaking up the cross-linking structure under low intensity ultrasound or HIFU. In order to verify our hypothesis and obtain a novel kind of reversible ultrasound responsive drug carrier, we prepared a novel kind of hybrid MSN by grafting sodium alginate (SA) polymer onto the MSN surface, which was further cross-linked by CaCl_2_ solution after cargo loading. Preparation and each step surface modification of MSN particles were fastidiously characterized. The hypothesis that COO^−^-Ca^2+^ coordination bonds are dynamically reversible was tested by the gel-sol transition of the SA-CaCl_2_ gels under ultrasound. The responsive cargo release pattern and the possible mechanism behind that were further investigated by using different two kinds of ultrasound (low intensity ultrasound or HIFU). Almost no drug release behavior under heating at 100°C suggested that the acoustic cavitation was the primary reasons of release. This research may provide an effective method to achieve an on-demand drug release pattern by using remote ultrasound stimulation and build up the frame for advancing future therapeutic applications.

## Experimental

### Materials

Cetyltrimethylammonium bromide (CTAB, 99.9%), tetraethyl orthosilicate (TEOS, 99.99%), sodium alginate (SA), and N-hydroxysuccinimide (NHS) were purchased from Aladdin (China). 3-aminopropyltriethoxysilane (APTES, 98%) was obtained from Adamas Reagent Co. Ltd. The model drug, Rhodamine B, and 1-ethyl-3-(3-(dimethylamino)- propyl) carbodiimide (EDC·HCl) were purchased from Best Reagent Co. Ltd (Chengdu, China). Methanol, ethanol, toluene, sodium hydroxide (NaOH), hydrochloric acid (HCl), ethyl acetate (EtOAc), and anhydrous calcium chloride (CaCl_2_) were all analytical chemicals supplied by Kelon Chemical Reagent Co. Ltd (Chengdu, China). Toluene was dried by refluxing in the presence of calcium hydride (CaH_2_) prior to use. All of the other regents were used as received.

### Synthesis of Mesoporous Silica Nanoparticles (MSN)

The preparation procedure was followed on a previously reported method with a little modification (Lee et al., 2010; Chang et al., 2013). Cetyltrimethylammonium bromide (CTAB, 1 g) was absolutely dissolved in 500 mL of deionized water (DI water) in a three-necked flask. Then, 2.0 M NaOH (3.5 mL) as the base catalyzer of the sol-gel reaction was added. Under the heating of water bath, the above mixture was mechanically stirred at 300 rpm for 2 h when the temperature raised to 70°C. Thereafter, tetraethyl orthosilicate (TEOS, 5 mL) and ethyl acetate (5 mL) were added sequentially at intervals of 1 min. The mixture was stirred for another 30 s and immediately stopped to keep at 70°C for 2 h. The solvent was removed by centrifugation and the precipitate was washed with a large amount of DI water and ethanol in turn. Finally dried the product under vacuum at 60°C overnight and collected before using.

In order to make MSN possess internal mesoporous structure, the reflux process with acid methanol solution was applied. The as-prepared particles (1.0 g) were extracted by refluxing in methanol (100 mL) and concentrated hydrochloric acid (1 mL) at 60°C for 6 h. After centrifugation and washing with DI water and ethanol three times, respectively, the template-removed mesoporous silica nanoparticles (MSN) were dried at 60°C in a vacuum overnight.

### Synthesis of Amino-Modified MSN (MSN-NH_2_)

As-synthesized MSN (1.0 g) and anhydrous toluene (75 mL) were placed into a round bottom flask which had been purged with high pure N_2_, and then 3-aminopropyltriethoxysilane (APTES, 4 mL) was added. This mixture was stirred under an inert atmosphere at 85°C for 24 h. The particles were separated by centrifugation and washed with anhydrous toluene and ethanol three times, respectively. The final product was obtained through drying under vacuum at 60°C overnight.

### Synthesis of Alginate-Grafted MSN (MSN-SA)

Firstly, a solution of sodium alginate (SA, 0.2%, w/v) at pH 5.0 was prepared, which was adjusted by adding 1 M HCl solution. Next, 1-ethyl-3-(3-(dimethylamino)- propyl) carbodiimide (EDC, 5.8 g) was dissolved in the above SA solution (50 mL) in a round-bottom flask and stirred at room temperature for a while, followed by the addition of MSN-NH_2_ particles (0.30 g) and N-hydroxysuccinimide (NHS, 3.5 g). After stirring the reaction mixture for 24 h at room temperature, the solution was centrifuged and washed with an excessive amount of DI water. The alginate-grafted MSN (MSN-SA) was dried in a vacuum oven at 60 °C overnight.

### Cargo Loading and Ionic Coordinated Cross-Linking via CaCl_2_

The model cargo, Rhodamine B (RhB), was loaded into the channels of MSN-SA by soaking the MSN-SA particles (40 mg) in an aqueous solution of RhB (24 mL, 3 mM) at room temperature. After stirring in the dark for 24 h, the saturated CaCl_2_ solution (50 mL) was added and the reaction solution was continuously stirred for another 24 h, leading to the cross-link reactions between Ca^2+^ and the COO^−^. The RhB-loaded, Ca^2+^ cross-linked MSN were separated by centrifugation and washed with DI water more than 3 times until the supernatant was nearly colorless to remove the unloaded cargoes, and the supernatant was collected for loading capacity calculation. Finally, the resulting materials (RhB@MSN-SA@Ca^2+^) were dried in a vacuum oven at 60°C overnight. The loading amount of RhB was quantitatively evaluated by UV-vis spectroscopy and calculated by the following equation with the collected supernatant:

$$\begin{array}{l} \text{Loading Capacity }=\\ \frac{initial\;weight\;of\;RhB-supernatant\;weight\;of\;RhB}{total\;weight\;of\;cargo\;loaded\;particles}\;\times \;100\% \end{array}$$

The Ca^2+^ cross-linked MSN without RhB loaded (MSN-SA@Ca^2+^) was prepared by the method as same as the previously mentioned.

### Cargo Release

The cargo release experiment from RhB@MSN-SA@Ca^2+^ nanocarriers was operated as follows: particles (5 mg) were soaked in 5 mL of Dulbecco's PBS (dPBS, pH 7.4, with an additional 15 mM Ca^2+^) and transferred into a dialysis bag (MWCO 3500), which was maintained in 45 mL of dPBS buffer and shaken at 37°C. To measure the concentration of the released cargo, 3 mL external dPBS buffer was withdrawn and tested by UV-vis spectroscopy, with replenishing subsequently the same volume of dPBS buffer to keep a constant release medium.

To study the ultrasound (US) responsiveness of RhB@MSN-SA@Ca^2+^ nanoparticles, the sample dispersion was exposed to low intensity US (20 kHz) or high intensity focused ultrasound (HIFU, 1,1 MHz). Two different types of ultrasound devices are depicted in Figure S1. Before applying US irradiation, the RhB@MSN-SA@Ca^2+^ (5 mg) was ultrasonicated widely in dPBS solution (1 mL) for a short time. In the case of HIFU exposure, the suspension was sealed in a dialysis bag (MWCO 3500), which was immersed in a custom-built glassware containing another dPBS solution (10 mL). At every point of studied time, 3 mL of outer releasing medium was withdrawn to test the cargo release amount by UV-vis and replenished with the equal volume of fresh dPBS. As to the low intensity US, the double jacketed beaker was used to hold the nanoparticle suspension and the outer layer is filled with condensed water (<26°C). After a certain irradiation period of US, the suspension was transferred into a dialysis bag and then processed in the same way as before. The release property of hybrid nanoparticles treated with low intensity ultrasound was compared with those treated under HIFU condition.

### Pre-experiment of Ultrasonic Reversible Responsiveness

For *in vitro* ultrasound stimulation experiments, the gel was obtained by dropwise adding 50 mM CaCl_2_ solution to a stirred sodium alginate (SA) solution in a bottle. The intensity and duration of low intensity ultrasound were 9.02 W/cm^2^ and 5 min at 20 kHz. Low intensity ultrasound was carried out in an environment with condensed water, whose temperature was kept <26°C. As to HIFU stimulation, the gel was formed in the mouth of a tube and then the tube was placed upside down on the focal of HIFU wave. The power and application time of HIFU was 40 W and 5 min at 1.1 MHz. HIFU irradiation was carried out in a water bath, so the influence of focal thermo effect on the cross-linked structure should be studied. After that, gels were kept under different heating conditions (70, 100, 120°C) for an hour as control experiments, observing whether gel-sol transformation occurred, to exclude the thermo effect of HIFU.

### *In vitro* Cytotoxicity Assay

The cytotoxicity of MSN-SA and MSN-SA@CaCl_2_ was assessed by the standard MTT assay using HeLa cells. Five replicates were set in each test group. Firstly, Hela cells were seeded into a 96-well plate and the cell density was adjusted to 6 × 10^3^ cells/well. After overnight incubation, the cells were incubated with different concentrations of MSN-SA and MSN-SA@CaCl_2_ for 48 h, and then 0.5 mg/mL MTT (200 μ L) solution was added to incubate each well for another 4 h at a constant temperature of 37°C and 5% CO_2_. Then, the supernatant was removed, and the obtained crystals were dissolved in dimethyl sulfoxide (150 μ L). After gently shaking in the dark for 10 min, the average absorbance was analyzed by a microplate reader (Rayto, Rt2100c) at a wavelength of 492 nm.

### Characterizations

Cargo release profiles were obtained by a Cary 60 UV-Vis spectrophotometer (Agilent, USA). Fourier transform infrared (FT-IR) analysis was operated on a Nicolet-560 spectrometer. The morphology and mesoporous structures were visualized using a scanning electron microscopy (SEM) on a Quanta 250 instrument (FEI Co. Ltd, USA) and transmission electron microscopy (TEM) on Tecnai G2 F20 S-TWIN (FEI Co. Ltd, USA), respectively. Zeta-potential was measured by a Zetasizer Nano-ZS (Malvern) at 25°C. N_2_ adsorption–desorption isotherms were obtained on an Autosorb-IQ2 Fully Automatic Analyzer. Thermogravimetric analysis (TGA) was carried out on a PerkinElmer TGA4000 from 100 to 800°C (10°C/min) in N_2_ atmosphere. Powder small-angle X-ray diffraction (XRD) measurements were implemented using an Empyrean powder diffractometer. X-ray photoelectron spectroscopy (XPS) was carried out with a KRATOS XSAM800 spectrometer.

## Results and Discussion

### Preparation and Characterization of Hybrid Mesoporous Silica Nanocomposites

**Figure 1:**
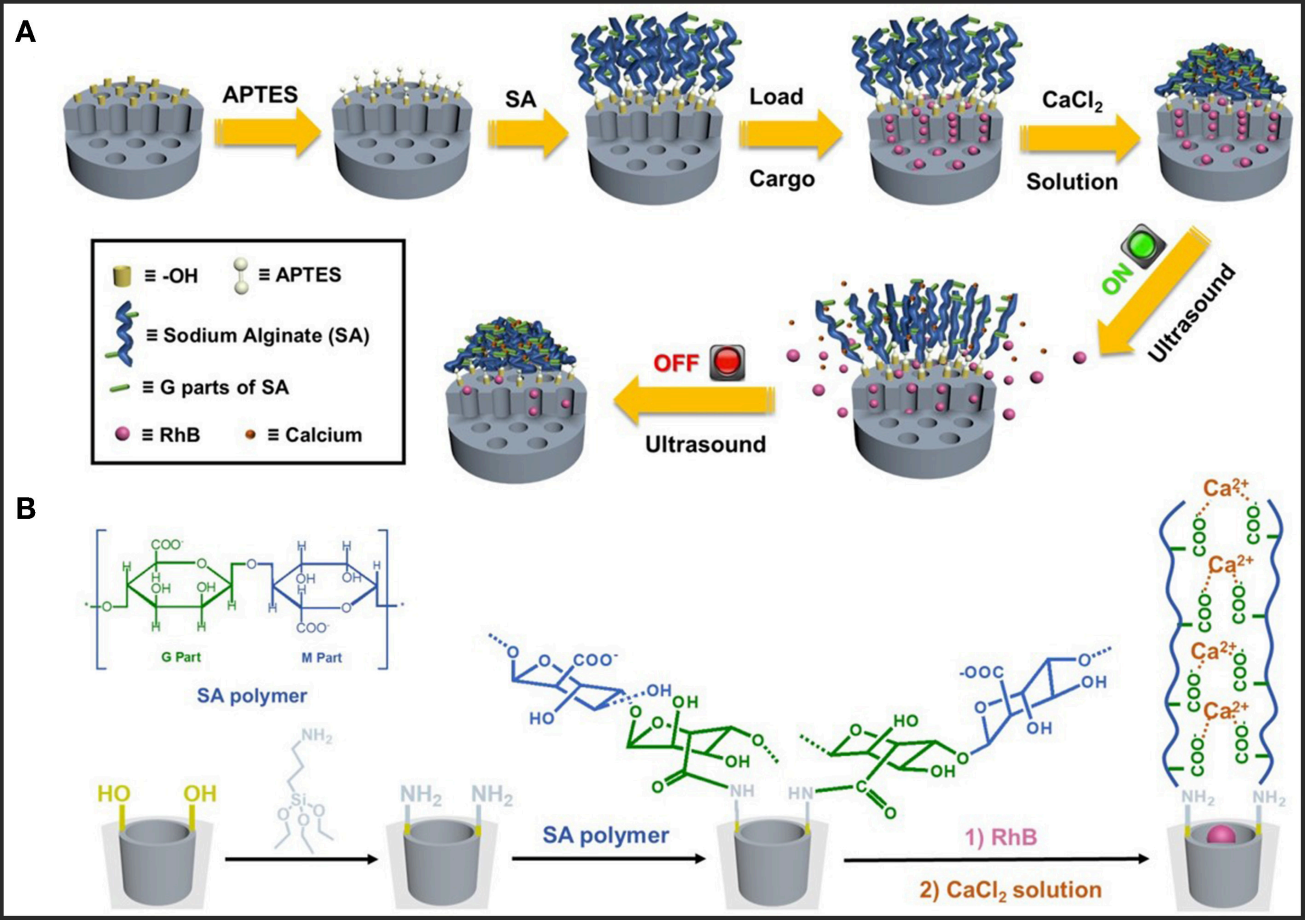
**(A)** Schematic illustration of the preparation process and ultrasonic reversible responsive behavior of the dynamic cross-linked network on hybrid MSN nanoparticles. **(B)** Molecular structure of sodium alginate (SA) and the synthetic route of calcium ion cross-linked MSN-SA.

**Figure 2:**
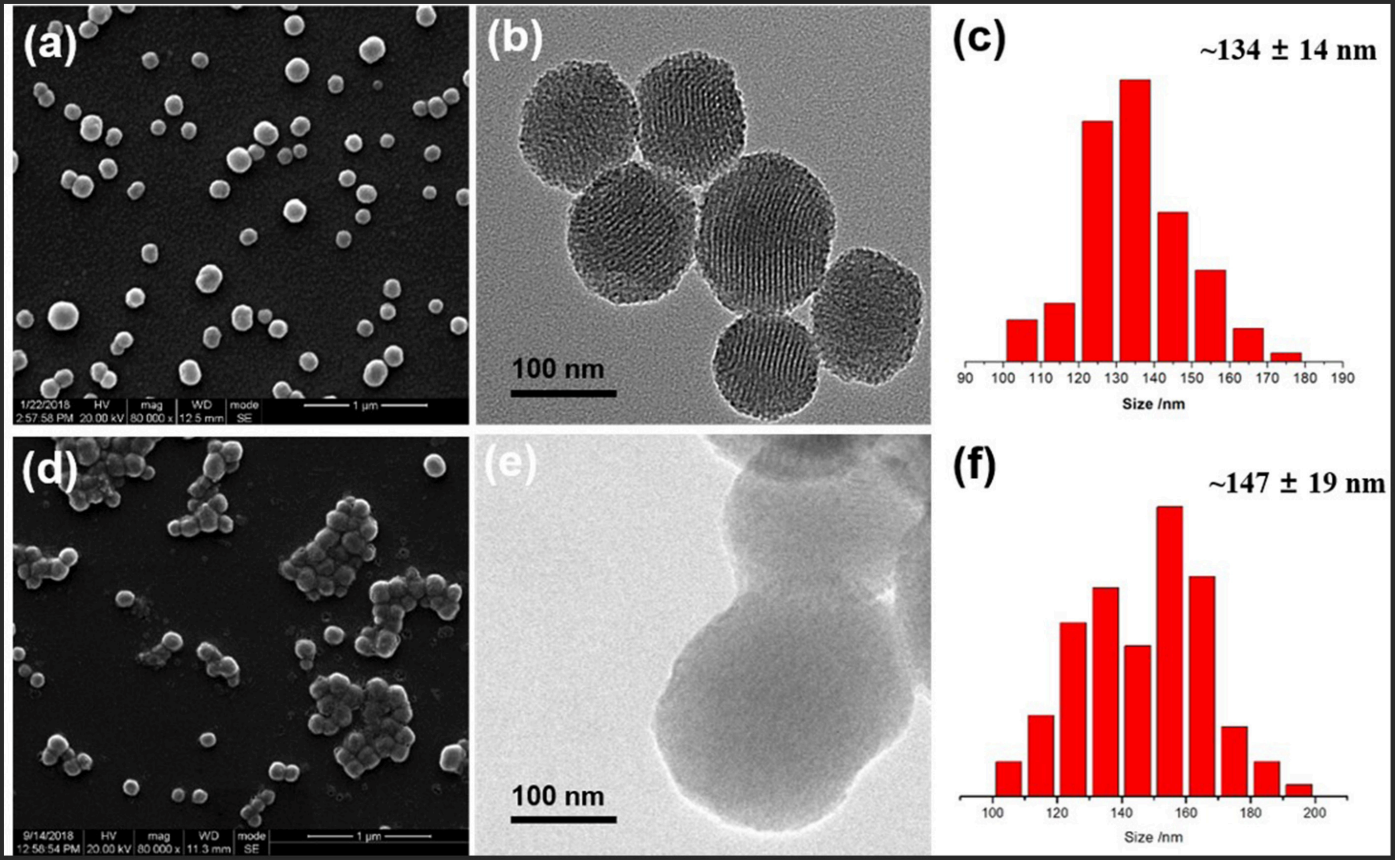
SEM images, TEM images, and size distribution of **(a–c)** MSN and **(d–f)** cross-linked MSN-SA@Ca^2+^, respectively.

Schematic illustration of our designed hybrid nanoparticles with reversible ultrasonic responsiveness and the preparation route were demonstrated in Figure 1. First, 3-aminopropyltriethoxysilane (APTES) was used to modify the MSN surface with -NH_2_ groups. Then, sodium alginate (SA) polymers were grafted onto the amino modified MSN (MSN-NH_2_), which was then cross-linked by CaCl_2_ solution to form the COO^−^-Ca^2+^ coordination bonds. As depicted in Figure 2, SEM and TEM images could intuitively confirm the particle morphology and pore structure. The pure MSN had a regular, spherical shape with an average diameter of 134 ± 14 nm, which was manually counted from 110 particles (Figures 2a,c). The channel structure of pure MSN could be clearly seen from the TEM image (Figure 2b). After a few steps of surface modifications, the average diameter of obtained particles increased to around 147 ± 19 nm (Figures 2d,f). The structure of channels became blurred and so did the edge of MSN materials which become less smooth (Figure 2e) proving that the polymer was successfully grafted and cross-linked on the surface of MSN.

**Figure 3:**
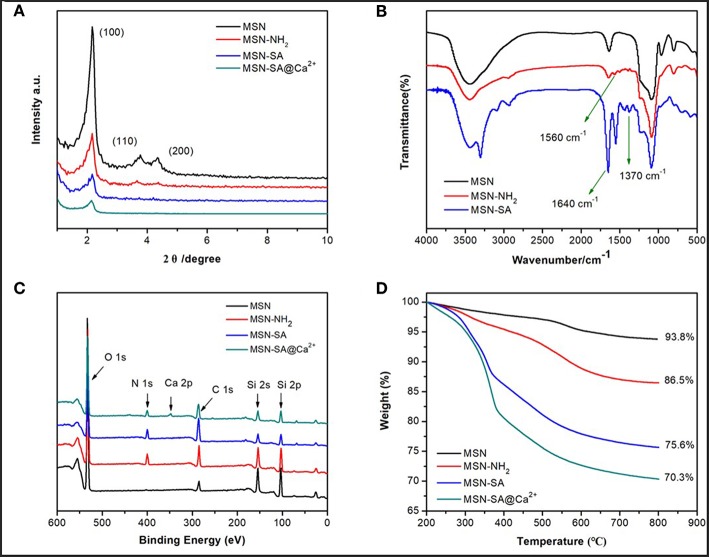
**(A)** Power small-angle XRD patterns of MSN, MSN-NH_2_, MSN-SA, and MSN-SA@Ca^2+^; **(B)** FTIR spectra of MCM (before CTAB template removal), MSN, MSN-NH_2_, and MSN-SA; **(C)** XPS spectra of MSN, MSN-NH_2_, MSN-SA, and MSN-SA@Ca^2+^; **(D)** TGA curves of MSN, MSN-NH_2_, MSN-SA, and MSN-SA@Ca^2+^.

The successful modifications of SA polymer onto the MSN surface were characterized by various spectroscopy means. Powder small-angle XRD analysis was employed to characterize the 2D hexagonal pore array structure of pure MSN, which was indicated as (100), (110), and (200) characteristic diffraction peaks (Figure 3A). After modifications, the three Bragg peaks of MSN-NH_2_ and MSN-SA showed a decreased intensity compared to the pure MSN curve, which resulted from the successful introduction of SA polymer on the surface of particles. Fourier transform infrared (FT-IR) spectroscopy was used to characterize each modification step performed on the surface of pure MSN, as displayed in Figure 3B. Firstly, the hydrocarbon vibrational peaks of the methyl and methylene groups at 2850 and 2920 cm^−1^ disappeared with the removal of the CTAB template, leaving a Si-O-Si vibration peak near 1100 cm^−1^ and a silicon hydroxyl peak near 3400 cm^−1^ which belonged to the neat MSN. A high intensity at 1630 cm^−1^ of MSN was ascribed to absorbed water bending vibration. Next, the amino groups (-NH_2_) were immobilized on the surface of MSN in the presence of 3-aminopropyltriethoxysilane (APTES), which was confirmed by the characteristic peak of MN-NH_2_ at 1560 cm^−1^. After the reaction between -NH_2_ and SA, two peaks at 1640 and 1370 cm^−1^ of MSN-SA assigned to the amide group (–CONH–) and carboxylic acid group (–COOH), respectively. This observation was proof that SA polymer had been successfully linked to the MSN surface, in line with the previous SEM, TEM, and XRD results (Figures 2, 3A).

To further verify the accomplishment of each step modification, the MSN-based samples were characterized by X-ray photoelectron (XPS) spectroscopy. As displayed in Figure 3C and Table S1, a new peak appeared at 404 eV ascribed to the N element from MSN-NH_2_ result (red curve in Figure 3C), which confirmed the successful reaction between silanol groups and APTES. The percentage of N element of MSN-NH_2_ was estimated to be 5%, whereas before modification only O, C, Si elements could be detected in the composition of MSN (black curve). Comparing the spectra of MSN-SA (blue curve in Figure 3C) with that of MSN-NH_2_ (red curve in Figure 3C), the intensity of C element increased, while the intensity of Si decreased due to the polymer shielding effect after SA polymer modification. The presence of Ca 2p peak at 347 eV of MSN-SA@Ca^2+^ spectra (green curve in Figure 3C), whose percentage of Ca element was calculated to be 2.23%, indicated the successful cross-linking of SA polymer by CaCl_2_ substance.

The weight loss percentage of various MSN-based samples was characterized by the thermogravimetric analysis (TGA), which were presented in Figure 3D. The final weight loss of MSN, MSN-NH_2_, MSN-SA, and MSN-SA@Ca^2+^ were 6.2, 13.5, 24.4 and 30%, respectively. Increasing weight losses indicated the successful immobilizations of APTES, SA polymer and cross-linked agent CaCl_2_ on MSN particles. There is about 4.56 mmol/g amino on MSN-NH_2_ as calculated from an extra 7.3% weight loss after being modified. The percentage of SA and CaCl_2_ on particles was about 11 and 5.6%, which further demonstrated the reaction of SA polymer and CaCl_2_. Beyond this, the average surface zeta potentials of all MSN-based materials were detected, and the data were shown in Figure S2 and Table S2. The zeta potential of MSN-NH_2_ was +38.09 mV, which indicated the introduction of amino groups compared with −27.56 mV of MSN. The zeta potential of MSN-SA was lowered to −34.10 mV since SA polymer has a large number of carboxyl groups. As to cross-linked nanoparticles, the zeta potential increased mainly due to the introduction of cationic calcium.

**Figure 4:**
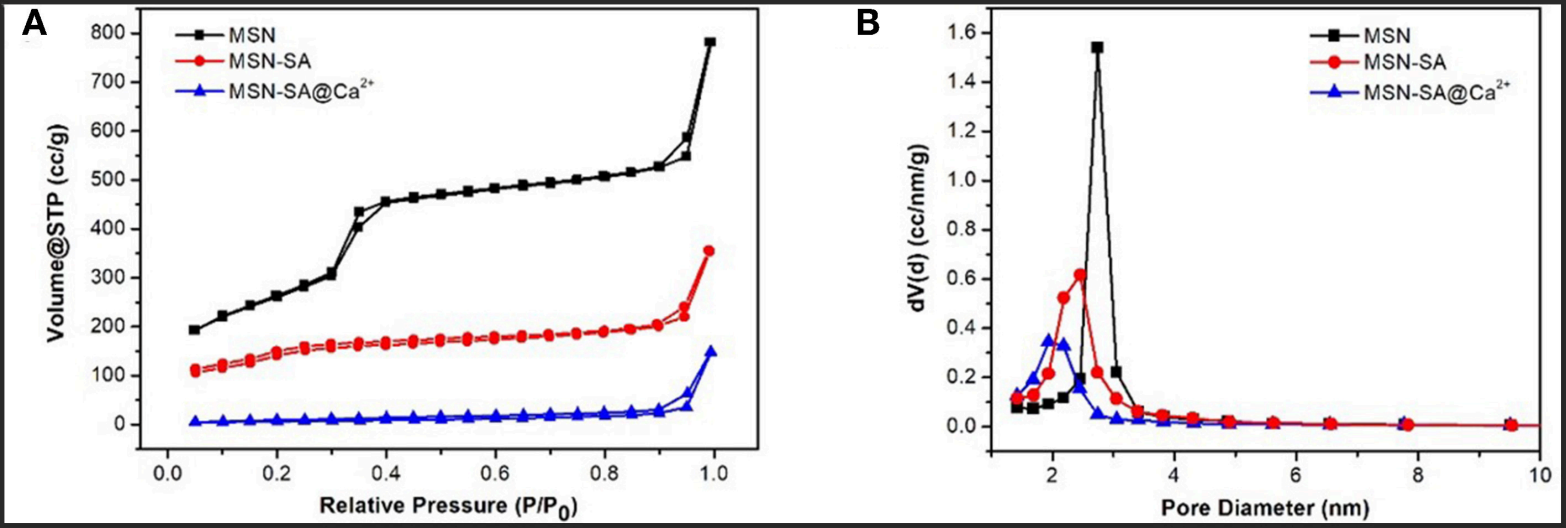
**(A)** Nitrogen adsorption-desorption isotherms and **(B)** pore size distribution of MSN, MSN-SA, and MSN-SA@Ca^2+^, respectively.

In addition, the surface area and pore size distribution of pure MSN and modified MSN nanoparticles were measured by the N_2_ adsorption-desorption isotherms (Figure 4 and Table 1). Clearly, the curve of pure MSN analyzed by the Brunauer–Emmett–Teller (BET) method showed a relatively sharp adsorption step at ~ 0.3 P/P_0_, which was classified as a typical IV behavior (Chang et al., 2013). This phenomenon was mainly due to the well-ordered mesoporous structure of MSN. It was worth noting that there were narrow H1-type hysteresis loops in the isotherms, which were mainly attributed to the capillary condensation. A narrow pore size distribution for each nanoparticle could be obtained by the Barrett–Joyner–Halenda (BJH) method. Nevertheless, the isotherms of MSN-SA and cross-linked MSN became flattered after modifications, and the pore diameter also decreased apparently. As summarized in Table 1, the surface area and the pore size of MSN was 970.42 m^2^/g and 2.74 nm, respectively. As to MSN-SA@Ca^2+^, these parameters were reduced to 252.36 m^2^/g and 1.93 nm, respectively, resulting from the successful surface modification of pure MSN which limited the absorption of N_2_.

**Table 1:** N_2_ adsorption-desorption results of MSN and hybrid MSN particles in brief.

**Sample**	**BET surface area (m^**2**^/g)**	**BJH pore size (nm)**
MSN	970.42	2.74
MSN-NH_2_	548.96	2.45
MSN-SA@Ca^2+^	252.36	1.93

### Exploration of Ultrasound Reversible-Responsiveness

Before investigating whether there was the ultrasonic reversible response of designed hybrid MSN materials, cross-linked SA-CaCl_2_ gel under ultrasound irradiation was studied first. According to the literature reported (Huebsch et al., 2014), ultrasound could break the complexation of divalent cations (e.g., Ca^2+^) and SA, while the presence of Ca^2+^ in physiological fluids would allow re-crosslinking after removal of the stimulus, therefore achieving a reversible on-demand response. To emphasize the role of ultrasound in the cross-linked network, two different intensity ultrasounds were applied and the reversible gel-sol transition behaviors were studied.

**Figure 5:**
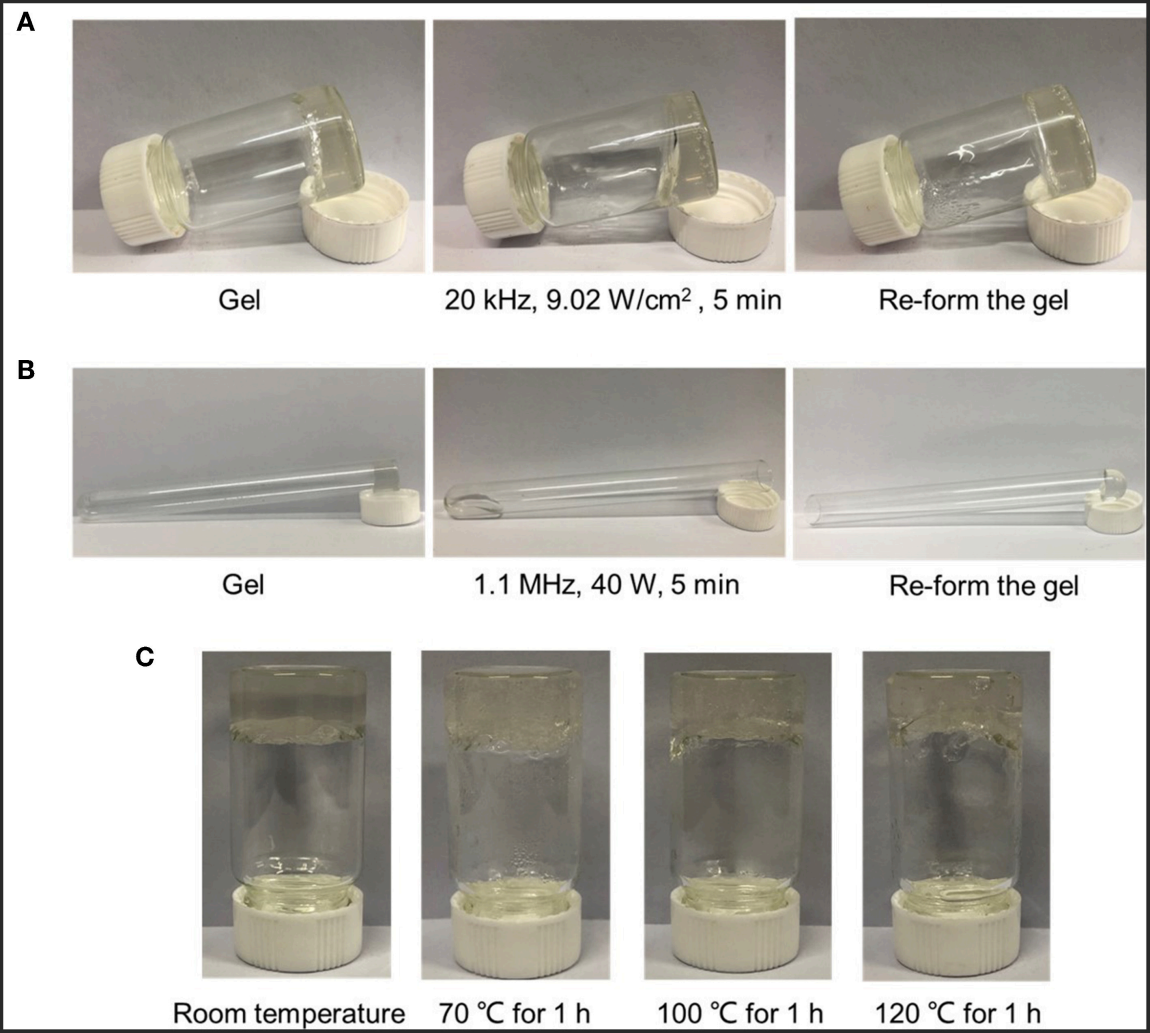
Ultrasound induced disruption and reformation of ionically cross-linked gels. Macroscopic images of the proposed ability of **(A)** low intensity ultrasound and **(B)** HIFU to induce temporary disruption of cross-linked structure by CaCl_2_ with SA polymer; **(C)** Macroscopic images of gels' status before and after treatment at different heating temperatures.

As shown in Figure 5, ultrasounds and heating conditions were applied to the collected gel, respectively. A series of macroscopic photographs clearly illustrated the reversible nature of the calcium-alginate cross-linking system and its response to the activation effect of ultrasounds. From Figures 5A,B, the original gel state would become a flowing liquid state after activating either by low intensity ultrasound or HIFU, which was named as a gel-sol transition. After removal of ultrasound, the sol state could subsequently return to the original gel state. Since the test of cross-linked gel stimulated by low intensity ultrasound was carried out in condensed water which the environmental temperature was maintained lower than 26°C, so the thermal effect could be eliminated and only the acoustic cavitation of low intensity ultrasound had an influence on the cross-linked structure. As to the HIFU, the thermal effect was stronger than that of low intensity ultrasound, so it was necessary to test which of the two effects was the main reason. A control experiment that gels were put into the different heating environment for an hour to test the main ultrasonic effect was designed. Figure 5C showed the images of SA-CaCl_2_ gel before and after thermal treatment. Only the content of water decreased and no gel-sol transition occurred. This phenomenon excluded the thermal effect from influence factors and concluded that the ultrasonic cavitation did play a major role in disrupting the ion-cross-linked structure of the gel (Paulusse and Sijbesma, 2004, 2008; Karthikeyan et al., 2008).

### Cargo Loading and Release

**Figure 6:**
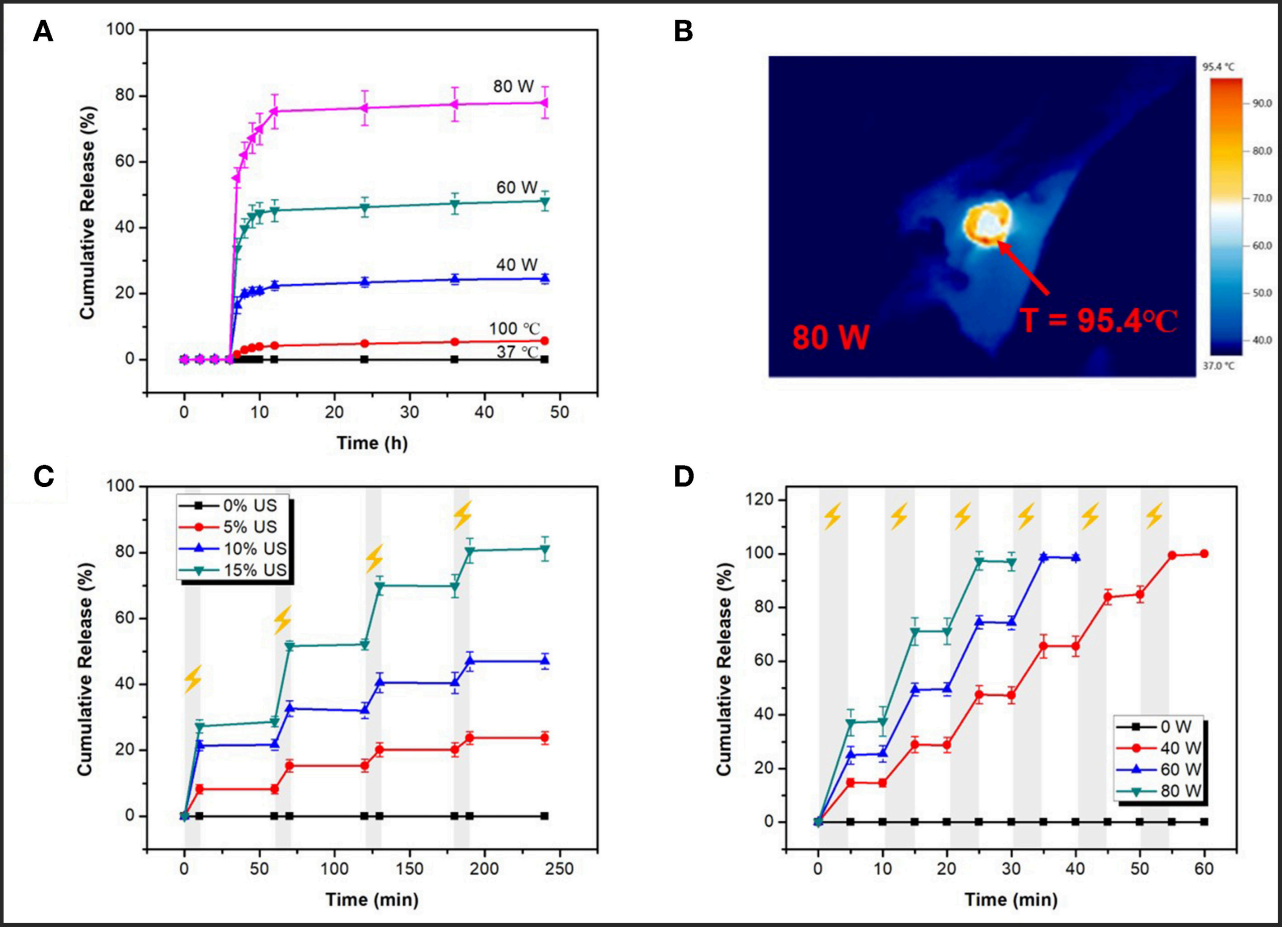
**(A)** Nature release profiles (release at 37°C) after different treatments for 5 min (40, 60, 80 W of HIFU and heating at 100°C); **(B)** The maximum temperature of latex membrane at the focal of HIFU wave recorded by an Infrared Thermal Imager at 80 W; **(C)** On-off releasing profiles at different outputs of low intensity ultrasound (0, 5, 10, and 15% of power, 10 min irradiation); **(D)** On-off releasing profiles at different power of HIFU (40, 60, 80 W, 5 min irradiation).

The feasibility of applying this ultrasonic reversible response ion-crosslinked structure to the on-demand cargo delivery was investigated by using Rhodamine B (RhB) as a model drug. There was about 14.3% of RhB loaded in the mesopores of MSN-SA, which was determined by a pre-established calibration curve (Figure S3A) using UV-vis spectroscopy. To investigate the US responsive release of cross-linked nanoparticles, the low intensity ultrasound and HIFU were both applied. Similarly, the releasing content of RhB was calculated using a standard calibration cure in Figure S3B. HIFU-triggered RhB releasing profiles under different power (40, 60, and 80 W) were firstly studied. For the first six hours, the sample was placed in the 37°C environment without irradiation. Immediately after 5 min of HIFU stimulation, the sample was continuously placed in the 37°C constant water bath shaker and the released cargo was collected at the specific time. As shown in Figure 6A, without stimulation, the cumulative release percentage was nearly zero for the initial several hours. However, a sudden increase in the release profile could be obtained by stimulating samples with HIFU. Upon exposure to HIFU, the percentage of final release of RhB could reach as high as 25, 48, and 78% at the output power of 40, 60, and 80 W, respectively. It has been demonstrated that the effects of ultrasound, both thermo effect and cavitation, could cause the sonodynamic shear to break the weak bonds, such as covalent bond, non-covalent π-π bond, metal coordination interaction, and hydrogen bond, therefore, damaging the integrity of structure network and discharge the embedded drugs (Li et al., 2010, 2018; Tong et al., 2013; Xia et al., 2016). In the previous part, thermo effects had been concluded that ould not make a major impact on the gel-sol transition of SA-CaCl_2_ gel (Figure 5C). It still needed to be determined for cross-linked MSN particles whether acoustic cavitation dominates the destruction of SA-Ca^2+^ cross-linked structure and the promotion of cargo release. The Infrared Thermal Imager was used to record the maximum temperature of latex membrane at the focal of the HIFU wave under different power outputs, the images were presented in Figure 6B and Figure S4. The temperature could reach to 61.3, 77.8, 95.4°C after irradiation for 5 min at 40, 60, and 80 W, respectively. 100°C, which was higher than the maximum temperature corresponding to the maximum power, was chosen to do the control experiment. After heating at 100°C for 5 min, the RhB releasing process was operated at 37°C, which was the same as the HIFU-triggered releasing experiment condition that was previously mentioned. In the end, the final percentage of RhB release was about 6%, which, although higher than that of untreated samples, was still much lower than that of HIFU exposed samples. These results further confirmed that cavitation effect rather than thermal effect of HIFU broke the SA-Ca^2+^ cross-linked structure and promoted the cargo release.

Figures 6C,D were cargo release profiles under the different power of low intensity ultrasound and HIFU. As depicted in Figure 6C, upon exposure to pulsatile ultrasound, cross-linked nanoparticles showed an obvious responsive release compared to non-stimulated samples (0% US). When ultrasound was turned on for 10 min, cargoes' releasing performance was apparent and when ultrasound was turned off, cargoes' releasing behavior stopped as well. Upon this pulsatile ultrasound (10 min-exposure per hour), the total RhB-releasing percentage could reach as high as 24, 47, and 81% at different powers (5%, 10%, 15% of total power) over 240 min. Then, the releasing profile was further studied under HIFU irradiation. A significantly increased release percentage was observed in an on/off pattern at a different output power of HIFU (Figure 6D). Ultrasonic stimulation was on for 5 min, then off for next 5 min, and nearly 100% of the RhB could be released in 60 min upon HIFU exposure. As an illustration, within a short duration of 55 min, the RhB-releasing percentage could reach up to 99% at the output power of 40 W. And yet it took only 30 min to obtain nearly 98% RhB-releasing at the output power of 80 W. All RhB cargoes could be released within 60 min upon HIFU exposure. What's important is that from the release pattern, the amount of RhB release was minor, even nil when either low intensity ultrasound or HIFU was off, proving from the side that the previously proposed SA-Ca^2+^ cross-linked structure indeed had the property of reversible self-recovery under ultrasound exposure. Comparing the time of releasing 80% of RhB, HIFU with no doubt had a shorter time (~22 min for 80 W) and, in other words, higher efficiency than low intensity ultrasound (~190 min for 15% of power) for the releasing stimulation. On the other hand, the maximum power of the above HIFU (80 W) was definitely safer than that of low intensity ultrasound (15% of total power) in practical human drug delivery because the latter would do harm to other healthy organizations in the body due to its strong cavitation effect. Thus, ultrasound, especially HIFU, could be an ideal external stimulation to realize a fast response releasing behavior with an on/off pattern, and these designed hybrid nanocarriers with reversible open/close responsiveness were intriguing candidates for effective drug delivery.

### *In vitro* Cytotoxicity Assay

**Figure 7:**
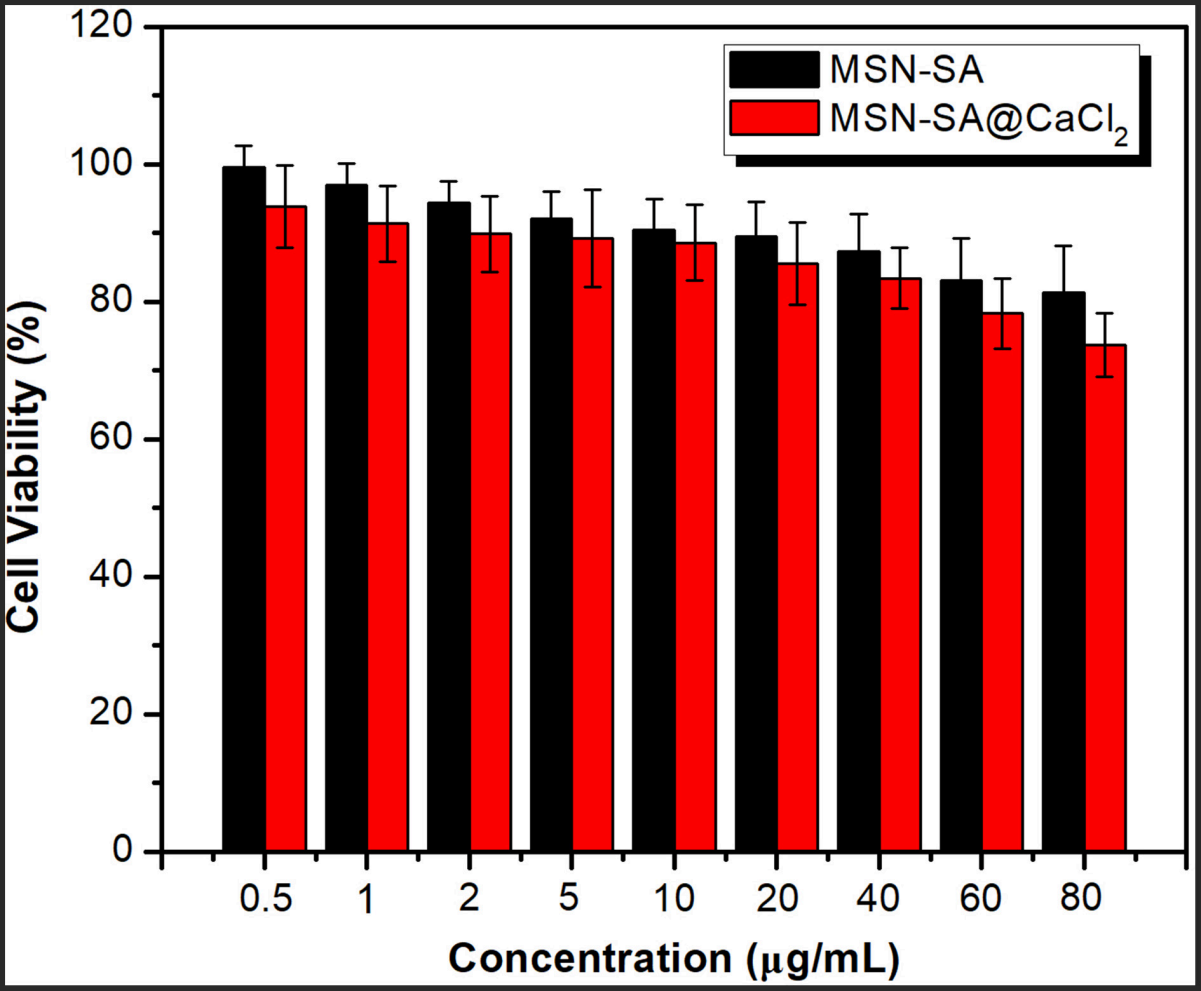
Cell viability of Hela cells in MSN-SA, MSN-SA@CaCl_2_ dispersion at different concentrations (0.5, 1, 2, 5, 10, 20, 40, 60, 80 μg/mL) by the standard MTT assay (*n* = 5).

The investigation on the cytotoxicity of blank hybrid nanoparticles of MSN-SA and MSN-SA@CaCl_2_ was also necessary before using them as drug carriers for future therapeutic application. The *in vitro* cytotoxicity experiment against HeLa cells was carried out with a standard MTT assay. As clearly illustrated in Figure 7, with the increase concentration of two nanoparticles, the cell viabilities performed a decrease trend. Nevertheless, MSN-SA and MSN-SA@ CaCl_2_ nanoparticles did not show high cytotoxicity even up to a concentration of 60 μg/mL, indicating these two nanoparticles both had good biocompatibilities and were suitable as drug carriers in therapeutic application.

## Conclusions

In summary, we developed a kind of hybrid organic-inorganic nanoparticles composed of MSN and SA-CaCl_2_ cross-linked coatings which possess a reversible ultrasonic responsiveness with an apparent on/off release pattern. The sodium alginate (SA) polymer was successfully grafted onto the surface of mesoporous silica nanoparticles (MSN) through an amino terminated silane coupling agent, then CaCl_2_ was introduced to cross-link the SA polymer layer which acted as a gatekeeper to prevent the RhB from premature releasing. The obtained hybrid nanoparticles presented a reversible-responsiveness to both low intensity ultrasound and high intensity focused ultrasound (HIFU), which was proved by calcium alginate gel-sol transformation experiments under ultrasound. The cargo loaded nanoparticles showed a fast ultrasound-induced release behavior and performed a good on/off release pattern under a pulsatile ultrasonic status. The mechanism of ultrasound-induced disruption was investigated and concluded that it was indeed the cavitation effect that dominated the process of breaking the metal coordination interaction. This study envisions that these new ultrasound stimulus reversible responsive organic-inorganic hybrid nanoparticles may possess potential applications in building on-demand drug delivery for timing-specific stimulation.

## Author Contributions

All authors listed have made a substantial, direct and intellectual contribution to the work, and approved it for publication.

### Conflict of Interest Statement

The authors declare that the research was conducted in the absence of any commercial or financial relationships that could be construed as a potential conflict of interest.
