# Supplementary material for: Ultrasound Reversible Response Nanocarrier Based on Sodium Alginate Modified Mesoporous Silica Nanoparticles
Source: Front Chem. 2019 Feb 11;7:59. doi: 10.3389/fchem.2019.00059 (PMC6378627; doi:10.3389/fchem.2019.00059)
Supplement: Supplementary file 1 [file Data_Sheet_1.pdf]

Supplementary Material

for

Ultrasound Reversible Response Nanocarrier Based on Sodium Alginate Modified Mesoporous Silica Nanoparticles

Xiaochong Li, Zhanhua Wang*, Hesheng Xia

State Key Laboratory of Polymer Materials Engineering, Polymer Research Institute, Sichuan University, Chengdu 610065, China.

E-mail: zhwangpoly@scu.edu.cn

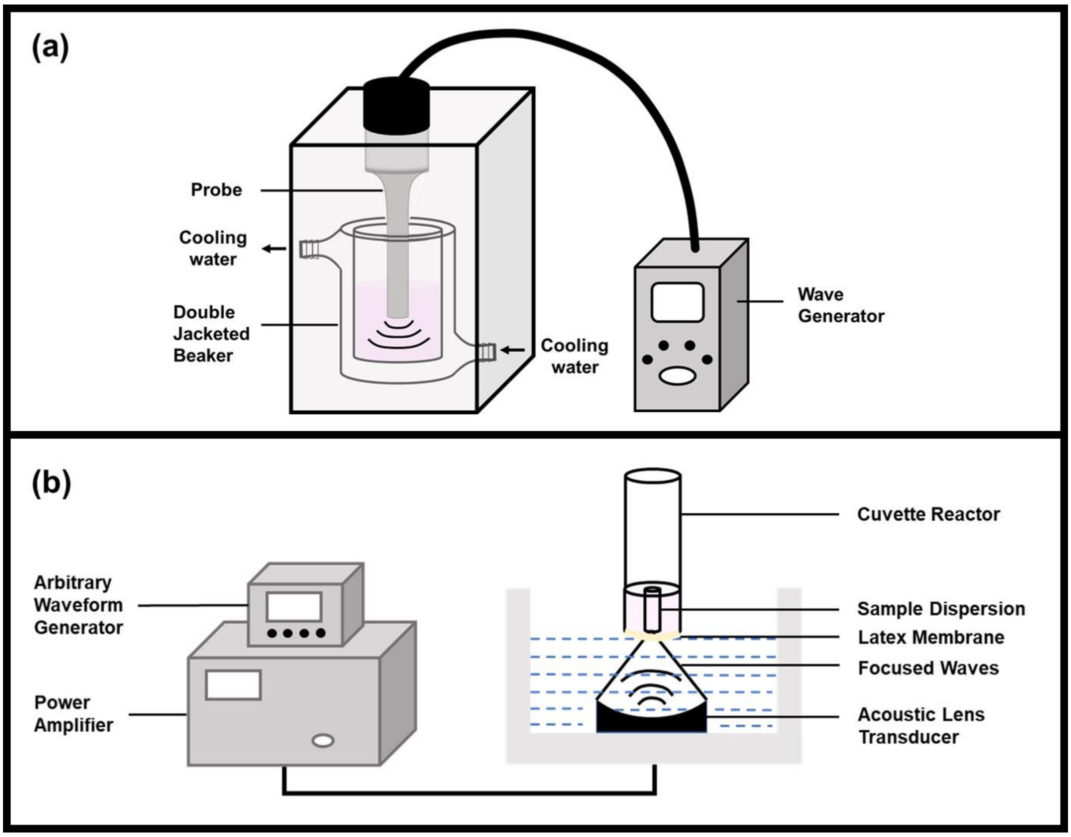

Figure S1. Schematic representation of two types of ultrasound devices used in this work: (a) low intensity ultrasound device (20 kHz, ~15 % of maximum output power); (b) high intensity focused ultrasound device (1.1 MHz, ~ 80 W).

Table S1. Percentage of different elements of samples calculated by XPS characterization.

AT%	C	O	Si	N	Ca
**MSN**	10.59	62.03	27.38	--	--
**MSN-NH_2**	21.33	49.43	24.25	4.99	--
**MSN-SA**	30.2	54.2	10.23	5.37	--
**MSN-SA@Ca^{2+}**	29.28	54.06	9.75	4.68	2.23

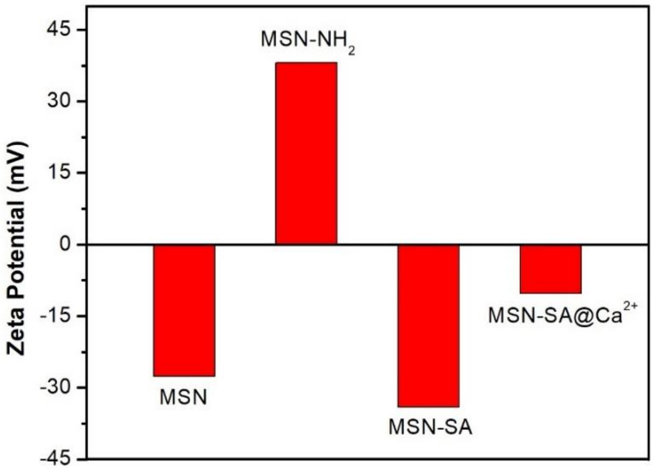

Figure S2. The Zeta potential of all MSN-based materials.

Table S2. The average Zeta potential of all MSN-based materials.

Materials	Average Zeta Potential (mV, n = 3)
**MSN**	-27.56
**MSN-NH2**	+38.09
**MSN-SA**	-34.10
**MSN-SA@Ca^2**	-10.26

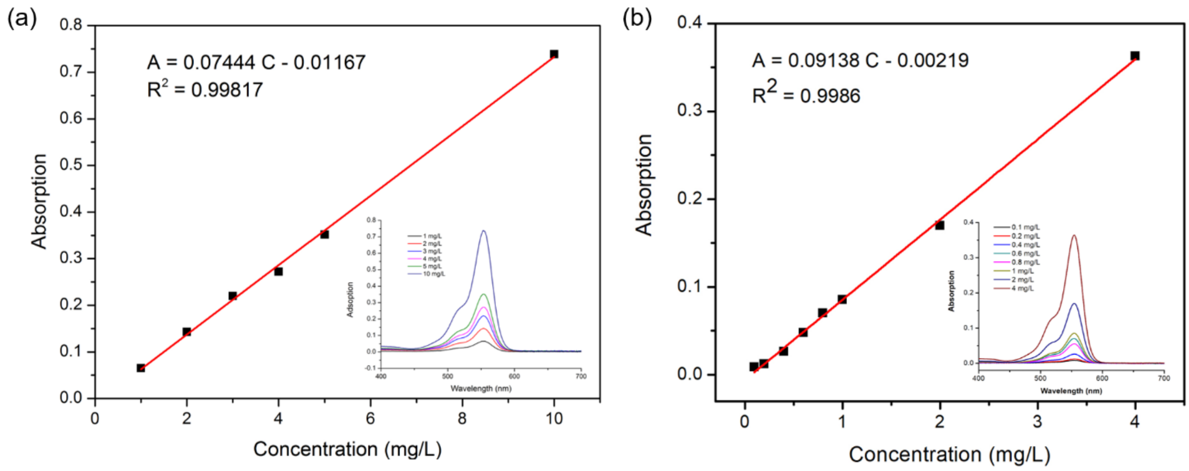

Figure S3. UV−vis calibration curve of (a) RhB aqueous solution and (b) RhB-dPBS solution;

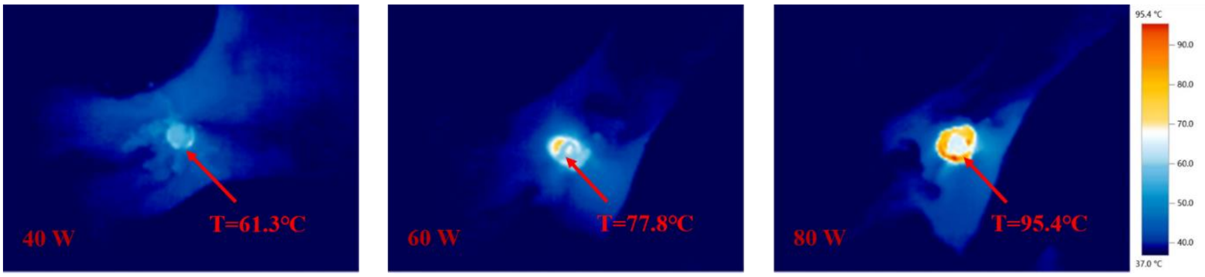

Figure S4. The maximum temperature of latex membrane at the focal of HIFU wave recorded by an Infrared Thermal Imager at different power outputs (40 W, 60 W and 80 W).

In order to test the focal temperature of HIFU at different output powers (40 W, 60 W, 80 W), the Infrared Thermal Imager was used to estimate. In order to keep constant with the experiment condition, the latex film was placed at the HIFU probe's focused place, then the HIFU stimulation was activated to stimulate for 5 min and simultaneously the Infrared Thermal Imager was started to record the temperature rising. From the results in Figure S4, it could be clearly seen that under different power outputs of HIFU, the focused points of waves had different maximum temperatures, and the temperatures increased with the increase of powers among them. Finally, the temperature 100 °C, which was more than the maximum temperature of 80 W irradiation for 5 min, was chosen to do the control thermal release experiment to investigate whether the thermal effect of HIFU had a major impact on cargo release.
